# Measurement of real pulsatile blood flow using X-ray PIV technique with CO_2_ microbubbles

**DOI:** 10.1038/srep08840

**Published:** 2015-03-06

**Authors:** Hanwook Park, Eunseop Yeom, Seung-Jun Seo, Jae-Hong Lim, Sang-Joon Lee

**Affiliations:** 1 Center for Biofluid and Biomimic Research, Department of Mechanical Engineering, Pohang University of Science and Technology (POSTECH), Pohang, 790-784, South Korea; 2 Industrial Technology Convergence Center, Pohang Accelerator Laboratory, POSTECH, Pohang, 790-784, South Korea

## Abstract

Synchrotron X-ray imaging technique has been used to investigate biofluid flows in a non-destructive manner. This study aims to investigate the feasibility of the X-ray PIV technique with CO_2_ microbubbles as flow tracer for measurement of pulsatile blood flows under *in vivo* conditions. The traceability of CO_2_ microbubbles in a pulsatile flow was demonstrated through *in vitro* experiment. A rat extracorporeal bypass loop was used by connecting a tube between the abdominal aorta and jugular vein of a rat to obtain hemodynamic information of actual pulsatile blood flows without changing the hemorheological properties. The decrease in image contrast of the surrounding tissue was also investigated for *in vivo* applications of the proposed technique. This technique could be used to accurately measure whole velocity field information of real pulsatile blood flows and has strong potential for hemodynamic diagnosis of cardiovascular diseases.

Circulatory disorders in vascular systems are one of the most serious causes of mortalities to date. Cardiovascular diseases are induced by various causes^1^; among which, wall shear stress (WSS) has been considered one of the most important parameter. The reduced and oscillating WSS in abnormal blood flows change the endothelial function and phenotype, which are closely related with the occurrence of atherosclerosis^2^. Considering that WSS could be calculated using the velocity gradient perpendicular to the vessel wall, velocity field measurement is essential for the estimation of WSS under *in vivo* conditions. Therefore, numerous studies have been conducted to measure blood flow and WSS under *in vivo* conditions^3^,^4^.

The micro-particle image velocimetry (PIV) technique has been widely utilized to obtain information on the velocity field of blood flows. However, the PIV technique could only be used to optically measure transparent flows and has limited penetration depth for opaque flows^3^,^5^,^6^. Recently, non-invasive imaging techniques, such as X-ray PIV^7^, magnetic resonance image (MRI)^8^,^9^, and echocardiography^10^,^11^ have been developed to measure opaque blood flows. A PIV measurement with high spatial resolution near the wall is required to accurately measure WSS^12^. Therefore, the synchrotron X-ray PIV technique with high spatial resolution was utilized in the present study to obtain hemodynamic information on opaque blood flows through a non-destructive manner^7^,^13^,^14^.

The performance of the X-ray PIV technique has been gradually improving for the last decade. However, several technological limitations of this technique make *in vivo* measurements of opaque biological flows difficult. Suitable tracer particles for X-ray PIV measurements of blood flows under *in vivo* conditions are difficult to fabricate because of their low bio-capability^14^,^15^, low image contrast under *in vivo* conditions^16^, and high agglomeration^17^. Although speckle patterns of red blood cells (RBCs) in X-ray images are used to obtain velocity field information on blood flows without tracer particles^18^, this approach is difficult to apply in *in vivo* measurements because of the low image contrast of X-ray images at high-speed. In addition, the surrounding tissues of the blood vessels significantly deteriorate the image blood flow contrast to a level at which measuring blood velocities without artificial tracer particles is impossible. Therefore, a new tracer particle that could confer high contrast in X-ray images is needed for *in vivo* measurement of real blood flows. Our research group focused on CO_2_ gas bubbles. CO_2_ gas with high negative contrast has been used as a contrast agent in clinical angiographic imaging^19^,^20^. The hypersensitive reaction of CO_2_ gas is relatively less than that of iodine contrast agent^21^. In addition, the intravascular supply of CO_2_ gas is unaffected by pH, pO_2_, and pCO_2_ levels. Although CO_2_ gas angiography poses several risks, the safe injection rate and suitable range of injection volume have already been reported^22^. Recently, our research group fabricated CO_2_ microbubbles using mechanical agitation and used them as flow tracers to obtain velocity information of blood flows under *in vitro* condition^23^.

The velocity fields of steady blood flows have been measured *in vitro* using CO_2_ microbubbles in our previous study^23^. However, hemorheological properties, such as RBC deformability and aggregation, may change during *in vitro* exposure of blood samples^24^. In addition, real blood flows measured *in vivo* exhibit pulsatile rather than steady flow behavior. The acceleration and deceleration of blood flows during a cardiac cycle influence the traceability of CO_2_ microbubbles. Therefore, the performance and accuracy of the X-ray PIV technique combined with CO_2_ microbubbles should be demonstrated under more reasonable pulsatile conditions. A rat extracorporeal bypass loop model was utilized because it circulates real blood with pulsatility through an external loop without hemorheological changes. This extracorporeal model is suitable for investigating the hemodynamic characteristics of pulsatile flows^25^,^26^.

In the present study, the feasibility of the X-ray PIV technique combined with CO_2_ microbubbles under *in vivo* conditions was assessed by applying this technique to *in situ* pulsatile blood flow. In addition, the effects of the surrounding tissues on the measurement performance of this technique were investigated. The velocity information obtained through the proposed X-ray PIV technique was compared with those obtained through an *in vitro* experiment using silver-coated hollow glass beads as tracer particles to check the traceability of the CO_2_ microbubbles under *in situ* conditions. In addition, the variations in the relative cross-correlation peaks were investigated according to the thickness of the surrounding tissues.

## Results

### Buoyancy effect of CO_2_ microbubbles

**Figure 1:**
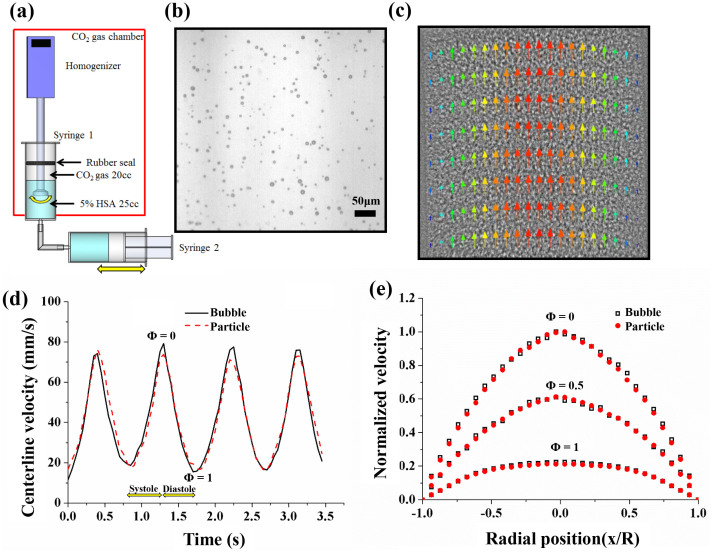
(a) Schematic of the experimental apparature for generation of CO_2_ microbubbles. (b) Optical image of CO_2_ microbubbles. (c) Instantaneous velocity field superimposed on the corresponding X-ray image of CO_2_ microbubbles flowing in a circular pipe. (d) Variations in the centerline velocities of microbubbles and silver-coated hollow particles in a Newtonian fluid flow with a pulsatile input frequency of 1.13 ± 0.04 Hz. (e) Normalized radial velocity profiles obtained using microbubbles and silver-coated hollow particles as flow tracers in a Newtonian fluid.

The CO_2_ microbubbles fabricated through mechanical agitation have a hollow structure (Figs. 1a–b). Their traceability under a given flow depends on the size of the CO_2_ microbubbles. This fact is closely related with the change in the buoyancy force caused by the density difference between the working fluid and CO_2_ gas. The mean diameter of the fabricated CO_2_ microbubble used in this study is approximately 13.3 μm. This size has been recommended in a previous study as optimal for velocity field measurements considering the interrogation window size of the PIV technique and the spatial resolution of captured X-ray images^23^. The terminal velocity of buoyant CO_2_ microbubbles could be obtained using a modified Stokes law^27^ given by
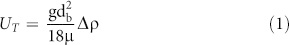
where *g* is the gravitational acceleration; *d_b_* is the diameter of a microbubble; *μ* is the fluid viscosity; and *Δρ* is the density difference. The terminal velocity caused by the buoyancy of the CO_2_ microbubbles was about 0.043 mm/s. This value is less than 0.5% of the average flow velocity of the working fluid supplied by a peristaltic pump. For a more accurate velocity assessment, the terminal velocity caused by the buoyancy effect is subtracted from the velocity field data obtained using X-ray PIV technique.

### Performance of CO_2_ microbubble as a flow tracer

To validate the use of CO_2_ microbubbles as suitable flow tracers for pulsatile blood flows, the measured velocity data of the CO_2_ microbubbles are compared with those of 14 μm silver-coated hollow glass beads widely used as tracer particles in X-ray PIV experiments. Figure 1c shows a typical instantaneous velocity field of a PBS solution seeded with CO_2_ microbubbles and the corresponding X-ray image. Variations in the centerline velocities measured with CO_2_ microbubbles and glass beads are compared in Fig. 1d. In the experiment, the pumping frequency is 1.13 ± 0.04 Hz. The centerline velocities measured by both tracer particles show good agreement. Φ = 0 and Φ = 1 are the end of the systole and diastole phases. To depict the periodic changes of velocity distributions in a pulsatile flow, the velocity profiles are normalized with the centerline velocity at the end of the systolic phase. Figure 1e shows that the normalized velocity distributions at three different phases (Φ = 0, 0.5, and 1) for both tracer particles are also well matched. The detailed flow characteristics are compared in Table 1.

**Figure 2:**
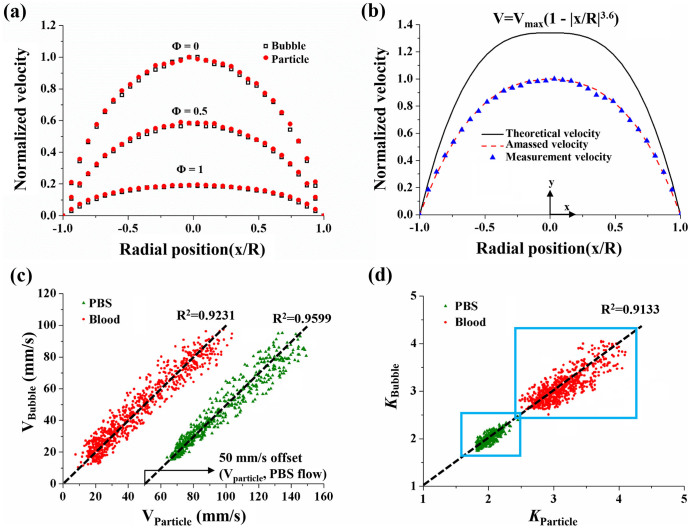
(a) Comparison of normalized velocity profiles obtained using CO_2_ microbubbles and silver-coated hollow glass particles in a blood flow with 40% hematocrit. (b) Normalized velocity at the end of diastolic phase Φ = 1. Solid and red dotted lines indicate the real and amassed velocity profiles, and blue triangles represent experimental results. (c) Scatter plots of the centerline velocities are measured using glass particles and CO_2_ microbubbles. Data set for PBS flow of 50 mm/s obtained using glass particles are shifted to evidently distinguish the two plots. (d) Scatter plot of the blunt indices for CO_2_ microbubbles(K_Bubble_) and silver-coated glass particles(K_Particle_). Linear regression line is included.

Figure 2a shows the normalized velocity profiles of the pulsatile blood flows at the phases of Φ = 0, 0.5, and 1. Although the pumping frequencies for the PBS and blood flows are identical, the general shape of the velocity profiles of blood flows is considerably different from that of the PBS solution (Figs. 1e and 2a). This phenomenon results from the shear thinning effect of the blood flow. The velocity profiles of the blood flows can be expressed as follows^28^
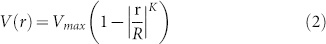
where *K* and *R* are the bluntness index and radius of a circular tube, respectively; *V_max_* is the centerline velocity; and *r* is the radial distance from the tube center. The *K*-value for a parabolic velocity profile is 2. When the *K*-value is larger than 2, the blood flow has a blunt velocity profile. Figure 2b shows a typical velocity profile obtained using X-ray PIV technique and the corresponding amassed velocity profile. Several mathematical formulas are applied to the velocity profile obtained using X-ray PIV technique to collect accurate velocity information on shear-thinning blood flows because all particles in the pathway of the X-ray beam propagation contribute in the evaluation of the cross-correlation coefficient for a PIV measurement^7^,^14^,^23^. The blue triangle indicates experimental data, and the red dot line represents amassed velocity profile. The real velocity profile (solid black line) is included for easy comparison.

Figure 2c shows a comparison of the centerline velocities of PBS and blood flows measured using CO_2_ microbubbles (V_Bubble_) and glass particles (V_Particle_). The dashed lines indicate linear-fitting curves. To easily compare the centerline velocities of PBS and blood flows, the horizontal axis is offset by 50 mm/s for the centerline velocity of the blood flow obtained using glass particles. The R^2^ values for the PBS solution and blood flows are 0.923 and 0.960, respectively. Figure 2d shows the variations in the *K*-values of the CO_2_ microbubbles (K_bubble_) and glass particles (K_Particle_). The green triangles indicate *K*-values of a Newtonian PBS solution, and the red circles denote *K*-values of the blood flow. The R^2^ value is 0.913, and the slope of the dashed line is 0.9347. To compare the centerline velocity and *K*-value between the CO_2_ microbubbles and glass particles, the corresponding data from10 different phases (Φ = 0, 0.11, 0.22. 1) are averaged over 16 cycles.

### Rat extracorporeal loop

**Figure 3:**
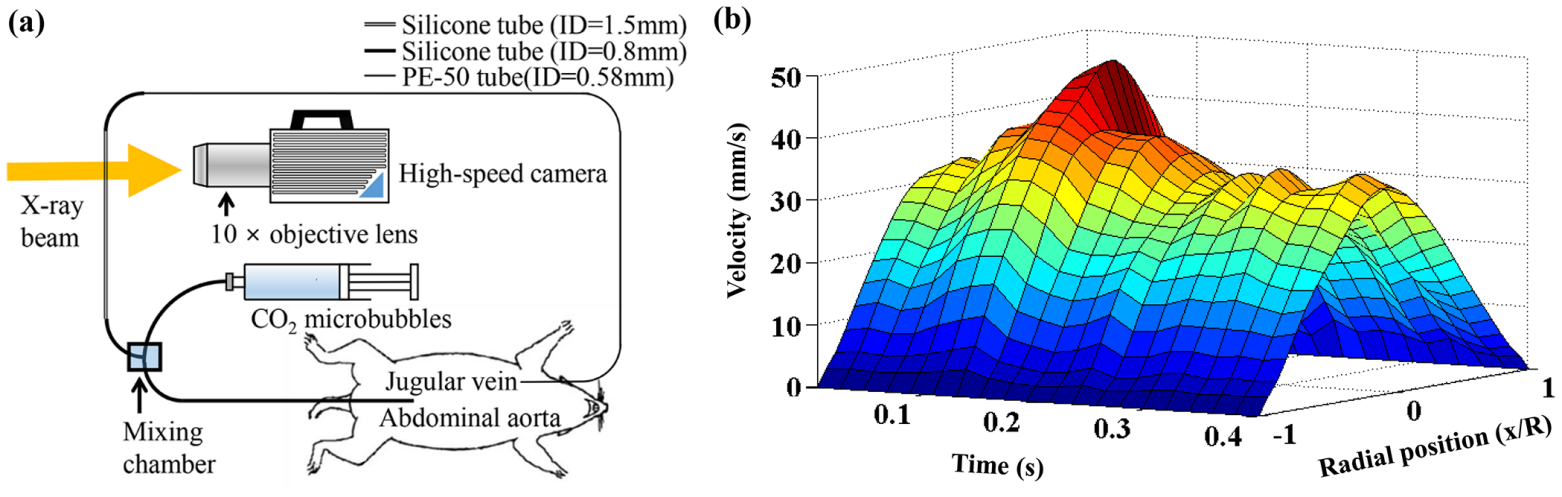
(a) Schematic of the rat extracorporeal loop system with a microbubble injection device. (b) Temporal variation of radial velocity profile in the rat extracorporeal loop in a cardiac cycle.

The velocity field information of pulsatile blood flows in the rat extracorporeal loop was acquired by using CO_2_ microbubble as tracer particles of the flow. The performance of the CO_2_ microbubbles in the X-ray PIV velocity field measurements was verified through *in vitro* experiments. Figure 3a shows a schematic diagram of the rat extracorporeal bypass loop system. Figure 3b shows temporal variations in the radial velocity profile of a cardiac cycle. The experimental data are collective average of seven cardiac cycles. The centerline velocity at the systolic and diastolic phases is 47.97 ± 1.98 and 23.19 ± 1.28 mm/s, respectively. The pulsatile index is 0.7720 ± 0.034. The mean frequency of the pulsatile blood flow is 2.53 Hz, and the mean *K*-value is 2.83 ± 0.161.

### Effects of surrounding tissues

To verify the feasibility of the proposed X-ray PIV technique combined with CO_2_ microbubbles under *in vivo* conditions, the effects of the surrounding-tissue thickness were investigated because these effects are crucial factors that deteriorate the image contrast of the CO_2_ microbubbles. The surrounding tissues were extracted from the pork neck and were placed in front of the test section. The blood flow seeded with CO_2_ microbubbles was measured under identical experimental conditions.

**Figure 4:**
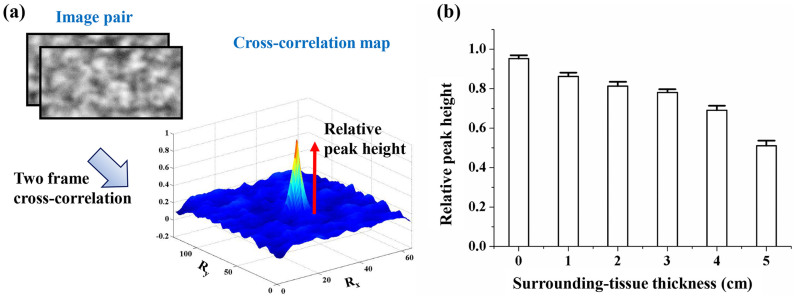
(a) Interrogation windows in two consecutive X-ray images and corresponding cross-correlation map when thickness of the surrounding tissues is 1 cm. (b) Relationship between the relative peak heights in cross-correlation maps and surrounding-tissue thickness.

Figure 4a shows two consecutive images, and the corresponding cross-correlation map when the surrounding-tissue thickness is 1 cm. The peak height in the cross-correlation map was used to investigate the signal intensity instead of the signal to noise ratio^28^. Figure 4b presents the relationship between the relative peak heights in the cross-correlation maps according to the thickness of the surrounding tissues. To assess the general trend, the relative peak height was determined by averaging 10 repeated experimental results. When no surrounding tissue is observed, the peak height has the highest value of 0.953 ± 0.016. The relative peak height decreases with increasing thickness of the surrounding tissues. The absorption and phase contrasts also decrease with increasing thickness of the surrounding tissues. When the thickness of the surrounding tissue is 5 cm, the relative peak height is about 0.511 ± 0.025.

## Discussion

The velocity information on opaque blood flows have been obtained with the aid of several non-invasive measurement techniques^7^,^9^,^10^. Among these non-invasive imaging techniques, the synchrotron X-ray imaging technique has been developed to measure blood flows^7^,^13^,^14^,^29^. In our recent study^23^, the feasibility of using CO_2_ microbubbles as flow tracers with high contrast to obtain velocity field information on blood flows was proven. In the present study, the traceability of the CO_2_ microbubbles in pulsatile blood flow was verified, and the hemodynamic features of pulsatile blood flows in a rat extracorporeal loop were experimentally determined. Figures 1 and 2 show that the velocity profiles obtained using CO_2_ microbubbles are well matched with those obtained using silver-coated hollow glass beads for both cases of Newtonian fluid and blood flows. The velocity profiles in the center region of the blood flows deteriorated because of the shear-thinning effect of blood^30^. To quantitatively analyze the shape changes in the velocity profiles, the *K* value model is adopted in the present study. The effect of hematocrit was checked, and the results are shown in Supplementary Fig. S1 and Table S1 because the bluntness of the velocity profiles and the traceability of the CO_2_ microbubbles are dependent on the blood hematocrit. A previous study^31^ has reported that the velocity profile becomes blunt as the hematocrit increases. Although the traceability of the CO_2_ microbubbles varies according to hematocrit, the variation is not extremely significant because the average flow rate is similar for all cases tested in the present study.

To further demonstrate the traceability and measurement performance of the CO_2_ microbubbles, the centerline velocity and *K*-value determined using the CO_2_ microbubbles are compared with those measured using silver-coated hollow particles, which are widely used in X-ray PIV experiments. As a result, the centerline velocity is well matched with the high R^2^ values. For a Newtonian PBS solution, the theoretical *K*-value in an ideal pipe flow is 2. However, the *K*-value slightly deviates from the ideal value in certain cases, especially in the acceleration and deceleration phases. Therefore, the estimated *K*-values for a Newtonian fluid are not exactly 2. For blood flows, the *K*-value varies depending on the centerline velocity and phase. The *K*-values measured using CO_2_ microbubbles and glass particles are highly correlated with the high R^2^ value (R^2^ = 0.9133).

Velocity information on pulsatile blood flow passing through the rat extracorporeal loop could be obtained using CO_2_ microbubbles. However, the contrast of the X-ray images is significantly reduced by the surrounding tissues of a biological sample under *in vivo* conditions. X-ray images are obtained based on the absorption and phase contrasts. The absorption contrast imaging method utilizes contrast difference caused by the different X-ray attenuation coefficients among samples. However, the absorption contrast in biological samples is usually low. Furthermore, the absorption contrast of tracer particles decreases because of the presence of surrounding tissues. Meanwhile, the phase-shift effect of X-ray beam propagation on the surrounding tissues of a biological sample is about 1000 times higher than the absorption contrast effect commonly used for clinical applications^32^. The phase contrast X-ray imaging technique utilizes reflections at the boundaries of a test object, and this technique is widely used in bio-medical applications^33^. The synchrotron X-ray imaging technique with coherent monochromatic X-ray beam is used to acquire phase contrast images. Kim et al.^34^ suggested the use of hollow-type microparticles for obtaining phase contrast X-ray images. Although CO_2_ microbubbles are phase contrast-based particles, the effects of the surrounding tissues still remain. The decrease in the image contrast of the CO_2_ microbubbles caused by surrounding tissues is demonstrated through the comparison of the relative correlation peak height with varying thicknesses of the surrounding tissues. When the relative peak height is larger than 0.5, the measurement accuracy of the X-ray PIV experiment is guaranteed^35^. Figure 4 shows that the measurement accuracy is guaranteed when the thickness of surrounding tissues is less than 5 cm. This tissue thickness can cover the thickness of the rat model.

In the present study, the traceability of the CO_2_ microbubbles, as flow-tracing particles in pulsatile blood flows, is demonstrated, and the velocity field information on real blood flow in the rat extracorporeal loop is obtained. The effects of the surrounding tissues are also investigated. The phase contrast X-ray PIV technique combined with CO_2_ microbubbles has strong potential in *in vivo* studies on hemodynamic characteristics. Nevertheless, the application of the synchrotron X-ray PIV technique for clinical diagnosis is difficult, because the use of a synchrotron facility is significantly limited and the energy flux of an X-ray beam is extremely high. The X-ray imaging experiments have dose limitation problems because of the high beam flux. Therefore, the proposed method would be used to investigate the blood flows of animal disease models at the initial stage. However, these technical limitations can be solved in the near future through the technological advances in X-ray imaging techniques^36^ by which phase contrast X-ray images can be acquired.

## Conclusion

In this study, the traceability of CO_2_ microbubbles in pulsatile blood flows was demonstrated by comparing the results with those obtained by using silver-coated hollow particles. Although the velocity profiles of the Newtonian fluid and shear thinning fluid flows are different, the results obtained using CO_2_ microbubbles and silver-coated hollow particles are in good agreement. The proposed X-ray PIV technique can be used to obtain hemodynamic information on rat blood flows using the rat extracorporeal loop system without any noticeable adverse effect. To check the diagnostic capability of the proposed technique, the effects of surrounding tissues on the contrast reduction are also investigated. The measurement accuracy is guaranteed when the thickness of the surrounding tissues is less than 5 cm. Although several problems have to be solved in advance for *in vivo* clinical applications, the X-ray PIV technique combined with CO_2_ microbubbles has strong potential for investigating the hemodynamic diagnosis of cardiovascular diseases.

## Methods

### X-ray imaging

**Figure 5:**
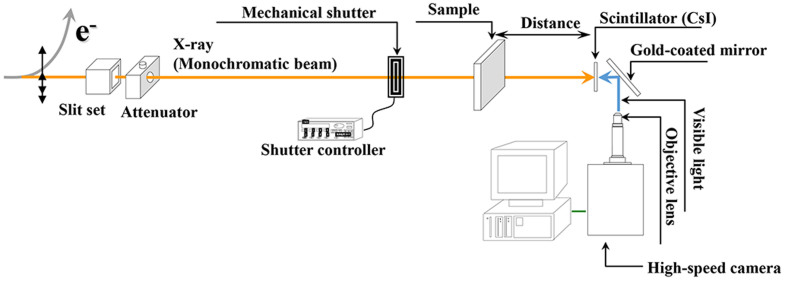
Schematic of X-ray PIV system established at the 6C beamline of PLS-II.

X-ray PIV experiment is performed at the 6C beamline of a Pohang Light Source (PLS-II). Figure 5 shows a schematic diagram of the X-ray PIV system. The beam current is 320 mA, and the storage energy of the synchrotron facility is 3 GeV. A monochromatic X-ray beam with beam flux of 1.2 × 10^12^ photon/s mm^2^ was used in this study. The median energy of the X-ray beam passing through a 1 mm-thick silicon wafer is 24 keV. The beam size is 8 mm (H) × 5 mm (V). The test sample is placed approximately 30 m downstream from the source. In general, the phase and absorption contrasts simultaneously occur in the X-ray images. To more clearly distinguish the biological samples, the phase contrast imaging is usually preferred compared with the absorption contrast imaging^37^. In this point of view, the phase contrast images of the CO_2_ microbubbles may be more suitable for tracing. Given that the distance from the sample to the detector is an important parameter in phase contrast images, this distance is fixed at 53 cm based on a preliminary test. As the X-ray beam passes through a CsI scintillator (500 μm thickness), the X-ray beam is converted to visible light. The X-ray images are consecutively recorded with a high-speed camera (SA 1.1, Photron, USA). The field of view is 1945 μm × 1945 μm (1024 pixels × 1024 pixels) under 10× magnification.

### Generation of CO_2_ microbubbles

Microbubbles have been successfully generated using mechanical agitation method^38^,^39^. In this study, CO_2_ microbubbles were also fabricated by mechanically agitating 5% human serum albumin (HSA) and CO_2_ gas (Fig. 1a). The 20% HSA was purchased from ChungShibJa Pharmacy Co., Ltd. (South Korea). The HSA medium is deionized water containing sodium chloride, sodium hydroxide, and acetyl-tryptophan. The 5% HSA is prepared by diluting 20% HSA in a PBS solution. Serum albumin is generally used as the encapsulating shell material of various ultrasound contrast agents^40^. CO_2_ microbubbles are generated using a homogenizer (IKA-T25 digital ULTRA-TURRAX, IKA, Germany) at 15,000 rpm for 7 min.

The direct injection of air bubbles into a circulatory vascular system induces a paradoxical air embolism^41^ related to acute limb ischemia and cerebrovascular accidents^42^. Therefore, CO_2_ microbubbles are generated in a CO_2_ gas chamber. The detailed generation procedures of microbubbles used in the present study are well described in a previous report^23^.

### *In vitro* experiment to validate the traceability of CO_2_ microbubbles

The working fluids used for *in vitro* experiments are PBS solution and blood with 40% hematocrit, which are Newtonian and shear thing fluids, respectively. The blood was supplied by Korea Red Cross Blood Services. The volume fraction of the microbubbles is 1.42%. The working fluid is supplied by a peristaltic pump (MP-1000, EYELA, Japan). The frequencies of the pulsatile flows are determined as 0.56 ± 0.03 and 1.13 ± 0.04 Hz. The corresponding average flow rates are 1.99 ± 0.07 and 2.51 ± 0.08 mL/min, respectively. The pulsating frequency was measured by counting the rotation speed of the peristaltic pump, and the average flow rate was determined using the weighing method. This measurement procedure was repeatedly performed for five times. The errors in the flow rate measurement were evaluated as 3.5% and 3.1% under two flow rate conditions.

### Preparation of a rat extracorporeal loop

Figure 3a shows a schematic diagram of the rat extracorporeal loop system. The extracorporeal bypass loop consists of a silicon tube (ID = 1.5 and 0.8 mm), and PE-50 tube (ID = 0.58, polyethylene tube). A mail Sprague-Dawley rat (12 weeks old, 354 g) was anesthetized with intramuscular injection of ketamine (100 mg/kg) and xylazine (10 mg/kg). The PE-50 tube at one end of the heparin-filled (10 IU/mL) bypass loop is cannulated into the right jugular vein. Subsequently, 500 IU/mL/kg heparin is injected into the right jugular vein to prevent blood coagulation inside the loop. Ten (10) min after heparin injection, a 22G catheter is inserted into the abdominal aorta. The silicon tube at the other end of the loop is then connected to the 22G catheter. Microbubbles were injected into the rat extracorporeal loop system at a flow rate of 0.1 mL/min using a syringe pump (PHD 2000, Havard apparatus, USA). All procedures performed on the animals were approved by the Animal Care and Ethics Committee of POSTECH and the methods were carried out in accordance with the approved guidelines.

### PIV measurement with digital image processing techniques

X-ray images are consecutively recorded at a frame rate of 1000 frames per second (fps) for 7.2 s. Velocity field information was obtained by applying a two-frame cross-correlation PIV algorithm to the captured X-ray images. The interrogation window size is 48 × 128 pixels with 50% overlapping. The inner diameter of tube is 1.5 mm.

To enhance the measurement accuracy, several digital image processing techniques are applied to the captured raw X-ray images before applying PIV algorithm. Given that X-ray image captured by a charge-coupled device camera contains the effect of beam fluctuations caused by electron beam instability, a flat field correction that eliminates background noise and spatial frequency filter is adopted in the present study^14^.

### Amassed velocity profile of blood flows

X-ray images contain information on all particles in the pathway of X-ray beam propagation. Therefore, X-ray images include 3D volumetric positional information on tracer particles. To extract 2D velocity field information from X-ray images, several mathematical formulas are used. For a Poiseuille flow in a circular pipe^14^, the ratio between the centerline velocities of the amassed velocity profile and real velocity is 2/3. However, the amassed velocity profile of the shear-thinning flows may be slightly different. For a blunt shear-thinning flow, the amassed velocity profile is modified by adopting a *K*-value model as depicted by^23^ (see Fig. 2b)
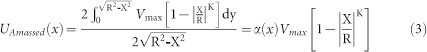
where *x* indicates the radial position. The relationship between tube coordinates *r*, *x*, and *y* is given by *r*^2^ = *x*^2^ + *y*^2^, where y-axis is the direction of the X-ray beam propagation. Considering that the *α* value depends on the radial position, a simulation of the Romberg integral is applied to obtain *α*. In this simulation, the radial position is uniformly divided into 6232 regions and the circular pipe is divided into 3.049 × 10^7^ segments. The theoretical amassed velocity profile and experimental results are iteratively curve-fitted with varying *K-*values. When the fitting velocity profile with a specific *K-*value has the largest R^2^ value based on the experimental results, it is selected as the amassed velocity profile.

## Supplementary Material

Supplementary Information Supplementary Information

## Figures and Tables

**Table 1:** Variations in hemodynamic charaicteristics of PBS solution and blood according to tracer particles and input frequency

	PBS				Blood			
	Silver-coated hollow particles		CO_2_ microbubbles		Silver-coated hollow particles		CO_2_ microbubbles	
Input frequency (Hz)	0.56 ± 0.03	1.13 ± 0.04	0.56 ± 0.03	1.13 ± 0.04	0.56 ± 0.03	1.13 ± 0.04	0.56 ± 0.03	1.13 ± 0.04
**Maximum velocity. (mm/s)**	59.27	95.05	61.84	97.88	52.68	79.12	53.40	81.57
**Frequency *f* (Hz)**	0.57	1.14	0.58	1.12	0.58	1.14	0.58	1.13
**Average flow rate (ml/min)**	1.95	2.51	2.03	2.53	1.97	2.53	0.96	2.55
