# Supplementary material for: Measurement of real pulsatile blood flow using X-ray PIV technique with CO2 microbubbles
Source: Sci Rep. 2015 Mar 6;5:8840. doi: 10.1038/srep08840 (PMC4351547; doi:10.1038/srep08840)
Supplement: Supplementary Information [file srep08840-s1.docx]

*Scientific report*

**Supplementary information**

**Measurement of real pulsatile blood flow using X-ray PIV technique with CO_2_ microbubbles**

Hanwook Park^a^, Eunseop Yeom^a^, Seung-Jun Seo^b^, Jae-Hong Lim^b^ and Sang-Joon Lee^a,*^

^a^ Center for Biofluid and Biomimic Research, Department of Mechanical Engineering, Pohang University of Science and Technology (POSTECH), Pohang, 790-784, South Korea.

^b^ Industrial Technology Convergence Center, Pohang Accelerator Laboratory, Pohang University of Science and Technology (POSTECH), Pohang, 790-784, South Korea.

* Corresponding author.

Tel.: +82 54 279 2169; Fax: +82 54 279 3199.

E-mail address: sjlee@postech.ac.kr (S.J. Lee)

**Supplementary**

**
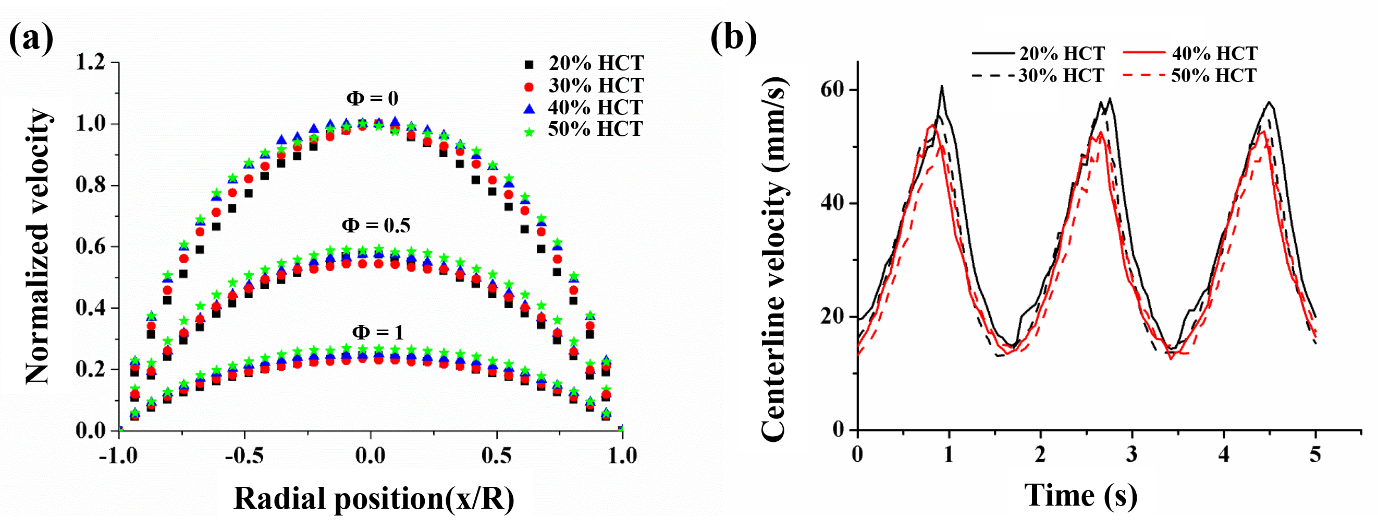
**

**Figure S1. (a)** Variation in normalized radial velocity profiles according to blood hematocrit. **(b)** Temporal variations in the centerline velocity of blood flow with different hematocrits.

| **RBC concentration** | **Average K-value** | **Maximum velocity (mm/s)** | **Average flow rate (ml/min)** |
| --- | --- | --- | --- |
| **20 %** | 2.51 | 60.66 | 1.96 |
| **30 %** | 2.81 | 56.65 | 2.03 |
| **40 %** | 3.15 | 53.40 | 1.94 |
| **50 %** | 3.65 | 51.70 | 2.04 |

**Table S1.** Variations in hemodynamic characteristics according to RBC concentration. As the RBC concentration increases, the normalized radial velocity becomes blunt in the center region. Variations in the average flow rates evaluated using velocity profiles are within the error level of the peristaltic pump.
